# Efficacy of a prevention program for eating disorders in schools: a cluster-randomized controlled trial

**DOI:** 10.1186/s12888-017-1454-4

**Published:** 2017-08-11

**Authors:** Antje Gumz, Angelika Weigel, Anne Daubmann, Karl Wegscheider, Georg Romer, Bernd Löwe

**Affiliations:** 1 Department of Psychosomatic Medicine and Psychotherapy, University Medical Center Hamburg-Eppendorf & Schön Clinic Hamburg Eilbek, Germany, Martinistraße 52, 20246 Hamburg, Germany; 2 Department of Medical Biometry and Epidemiology, University Medical Center Hamburg-Eppendorf, Germany, Martinistraße 52, 20246 Hamburg, Germany; 3 Berlin University of Psychology, Am Köllnischen Park 2, 10179 Berlin, Germany; 4 Department of Children and Adolescent Psychiatry, Psychosomatics and Psychotherapy, University Medical Center Münster, Germany, Schmeddingstr. 50, 48149 Münster, Germany

**Keywords:** Eating disorders, Prevention, Adolescents, Randomized controlled trial, Risk factors

## Abstract

**Background:**

Previous prevention programs in the school context have not addressed both genders, have been time-consuming, or have had deficits in the evaluation method. The aim of the present study was to evaluate the impact of a universal prevention program for female and male adolescents on eating disorder pathology and related risk factors.

**Methods:**

Between February 2012 and July 2014, 2515 students in 23 schools from 8th or 11th grade were assessed for eligibility in this longitudinal cluster-randomized controlled trial with a six months follow-up. Of those students, 2342 were cluster-randomized to the intervention condition which received a six school hours universal prevention program or to the no treatment control condition.

**Results:**

The complete case population comprised 724 students in the intervention (54.3% female, *M* = 14.3 years, *SD* = 1.61) and 728 in the control condition (57.0% female, *M* = 14.7 years, *SD* = 1.63). Random-effects analysis of covariance on the primary outcome showed no significant differences between the intervention and control groups in their eating disorder pathology change scores six months after the intervention. Regarding secondary outcomes, participants in the intervention group showed a greater increase in knowledge about eating disorders both after the intervention (*p* < .001, *ES* = 1.06) and six months later (*p* = .01, *ES* = 0.40). Greater reductions in anxiety severity were observed in the intervention group post-intervention (*p* = .02, *ES* = 0.22) which was not maintained at the six months follow-up. Results differed between participants from grade 8 and 11.

**Conclusion:**

The present universal prevention program can be particularly recommended for adolescents from grade 11.

**Trial Registration:**

ISRCTN 97989348

**Electronic supplementary material:**

The online version of this article (doi:10.1186/s12888-017-1454-4) contains supplementary material, which is available to authorized users.

## Background

Adolescence is a vulnerable period regarding the development of an eating disorder [1]. The prevalence rates for this age are 2% for anorexia nervosa, 1% for bulimia nervosa and 2% for binge eating disorder [1]. Substantially more adolescents are affected by eating disorder symptomatology, i.e., dieting, weight loss behaviors [2, 3] or restrained eating and weight concerns [4]. Once full-blown eating disorders emerge, the initiation of a recommended treatment is often delated [5] and particularly anorexia nervosa exhibits a high risk of chronicity [6]. Against this background, an implementation of eating disorder prevention programs during adolescence is particularly important [7].

Different considerations plead for an implementation of eating disorder prevention programs in the classroom setting during adolescence. From a theoretical point of view, female and male adolescents make up an important part of the social environment for each other [8]. Including complete classes as the most relevant peer group provides the opportunity to increase awareness of the adverse effect of peer pressure on eating disorder development [9]. Peers influence the extent of pressure to suit Western beauty ideals, individual appearance related attitudes and body dissatisfaction which are all risk factors for eating disorders [10, 11]. Additionally, an overlap of further eating disorder risk factors such as a low self-esteem and negative affects like depression and anxiety has to be expected in female and male adolescents [10, 11]. From a practical point of view, it is easier for schools to deliver prevention programs to whole classes as most schools are co-educational.

Prevention programs delivered to whole classes follow a universal prevention approach as female and male adolescents participate without prior screening and regardless of risk status. In the field of eating disorders, the efficacy of universal prevention programs in terms of effect sizes is typically lower than in selective approaches [12] which has been attributed to different baseline scores in unselected samples [13]. Bailey and colleagues summarized the results of 46 trials of universal prevention programs for eating disorders as well as six meta-analytic reviews [14]. Satisfactory effect sizes were observed for outcomes related to eating disorder knowledge while risk factor related outcomes were barely improved. The authors concluded that research opportunities for universal eating disorder prevention could be found in following a cognitive dissonance rather than a psychoeducational approach and by offering interactive interventions in a multi-session format. Another narrative review on effective eating disorder prevention programs underlined the need for universal prevention programs that address both genders [15].

A most recent randomized controlled trial compared three eight lesson universal prevention programs that addressed females and males with an average age of 13 years and aimed at reducing both eating disorder and obesity risk factors [16]. In one condition most encouraging findings were observed with regard to risk factor reduction in female and male participants (i.e. Media Smart). However, there was evidence of iatrogenic effects of the other two conditions (i.e. Life Smart, HELPP). In these conditions, the authors observed a significant increase of eating concerns and perceived pressure to be thin at the six months follow-up in female participants relative to the control condition (i.e. HELPP) and worsened scores on four eating disorder risk factor variables in female participants as well as worsened scores on three eating disorder risk factor variables in male participants (i.e. Life Smart).

Despite the promising evidence of some universal prevention programs most of these programs do not manage to be transferred from research into classroom practice on the long run [17]. Current universal prevention programs for eating disorders suffer from several shortcomings. A duration of six to nine weekly lessons is too long to suit the demands of school curricula for each school year. In other cases, scientific evaluations of the programs do not satisfy methodological standards regarding sample size [18, 19], randomization [20] and follow-up assessments [21, 22]. Furthermore, in most instances to date, and with some notable expectations (e.g. [23]) stakeholders including professional organizations that are in regular contact with schools are not yet sufficiently involved in the program development, implementation and dissemination to improve sustainability [24]. Thus, methodologically rigorous efficacy trials of short universal prevention programs which address female and male adolescents and fit the school curricular are urgently needed [13].

Against this background, this cluster-randomized controlled trial was designed to evaluate the impact of a brief universal prevention program on eating disorder pathology and related risk factors for female and male adolescents. We hypothesized that students who participated in the program would exhibit a significantly greater decrease in eating disorder pathology six months after participation than students who did not participate (primary outcome). We further expected this group difference to emerge directly after participation. Additionally, we hypothesized that students who participated in the intervention would more extensively increase their knowledge of eating disorders, show greater improvements in their self-concept, exhibit less internalization of Western beauty ideals, and report reduced levels of depression and anxiety compared with the outcomes for the control group both immediately after and six months after the intervention. Finally, we performed mediation analyses and subgroup analyses to describe the intervention’s effect in subgroups based on age and sex.

## Methods

### Study design and participants

The efficacy of the present eating disorder pathology reduction program was evaluated using a cluster-randomized controlled trial design.

All 144 secondary schools in the city of Hamburg, Germany, were invited to participate. Of those, ten schools actively refused to participate, and 111 did not respond to contact attempts. Thus, participants were recruited from *n* = 23 secondary schools between February 2012 and July 2014. Eligible participants were students in the 8th or 11th grades (age 14 and 17 years) who provided written informed consent. Additional written informed consent from a parent or legal guardian was required for participants under 16 years of age. Insufficient knowledge of German language was the only exclusion criterion.

The study was approved by the Ethics Committee of the Psychotherapist Chamber of Hamburg, Germany (July 26, 2011), as well as the Hamburg supervisory school authority. The study protocol has been published (see [25] or Additional file 1 available online), and the study was registered with the ISRCTN registry (ISRCTN 97989348).

### Randomization

Participating schools were randomized into the study conditions (intervention vs. control) according to the order of application. Randomization was conducted by an external institution (Department of Medical Biometry and Epidemiology, University Medical Center Hamburg-Eppendorf). Random allocation was performed in a 1:1 ratio stratified by school type (district vs. secondary school) and the socio-economic status of the school environment without blocking. During recruitment, several individual schools were combined with each other due to changes in the Hamburgian school system. As a consequence, the standardized socio-economic indicators of the individual schools were no longer applicable. Thus, random allocation had to be revised, and the result was an imbalance of schools in the study conditions. Once assigned, each school stayed in the same study condition for the entire study duration.

Before providing written informed consent, students and their parents or legal guardians obtained a letter of consent with a detailed description of the allocated study condition. Thus, participants could not be blinded to their study condition.

Participants in the two conditions received neither incentives nor feedback about individual test results. However, participants received a flyer with counselling and assistance services and were given the opportunity to contact a psychotherapist from the research team in case of any mental health issues.

### Outcome measures

There were three times of assessment: 1) one month prior to the intervention (baseline), 2) directly after the intervention (post), and 3) six months after the intervention (follow-up). Parallel time points were used for assessments in the control group. At all times of assessment, outcomes were assessed using a questionnaire set with self-report items. At baseline, demographic characteristics and body mass index (BMI) were assessed.

The primary outcome was changes in eating disorder pathology from baseline to the six months follow-up. Following the suggestions by Wilksch [17] the global mean score of the children’s version of the Eating Disorder Examination Questionnaire (Ch-EDE-Q; [26]) was used to assess the primary outcome. The Ch-EDE-Q total score is calculated from the mean score of 22 items which refer to eating disorder symptomatology occurrence in the past 28 days (range 0–6, with greater scores indicating more frequent experience of symptoms). The criterion validity of this questionnaire has been demonstrated in multiple studies and languages and there is empirical support from community-based samples for the temporal stability of the EDE-Q subscale scores over five to fourteen months [27]. The inner consistency of the Ch-EDE-Q global mean score in the present sample was Cronbach’s α = .955.

The secondary outcome scales included a questionnaire to measure *knowledge about eating disorders* (20 questions covering different aspects of eating disorder risk factors and symptoms, with response options including “right”, “wrong” and “don’t know”; range 0–20). The *Sociocultural Attitudes Towards Appearance Questionnaire* (SATAQ-G*;* [28]) was used to measure three aspects of the internalization of Western beauty ideals. The psychometric properties of this gender-adapted questionnaire have been established in adolescent samples of studies in different countries [29, 30]. The inner consistencies of the SATAQ-G subscales ranged between Cronbach’s α = .822 (awareness subscale) to .900 (internalization subscale). The *Multidimensional Self-Concept Scale* (MSCS; [31, 32]) was used to measure self-concept. In the present sample Cronbach’s α was .921. The well-validated *Patient Health Questionnaire-9* (PHQ-9*;* [33, 34]) and *Generalized Anxiety Disorder Scale* (GAD-7; [35, 36]) were used to measure the severity of depression and anxiety symptoms. Inner consistencies of these instruments were Cronbach’s α = .819 (PHQ-9) and α = .860 (GAD-7) in the present sample.

### Intervention

**Fig. 1:**
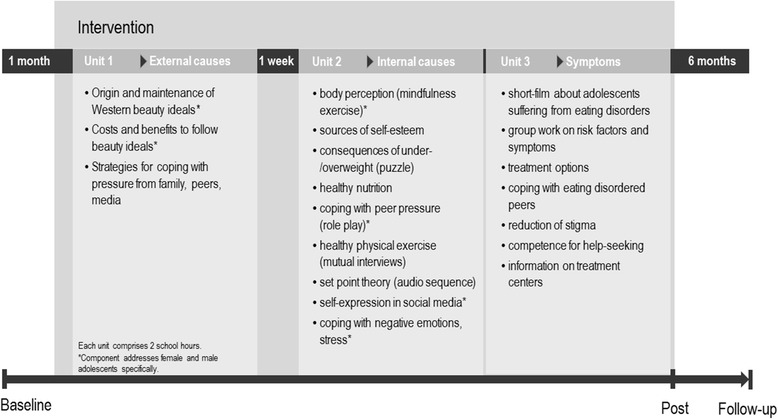
Overview of the intervention and study procedure. *Note*. Questionnaire-set baseline: grade, body mass index; parental educational level; immigrant background; Questionnaire-set baseline, post, follow-up: Children’s Eating Disorder Examination Questionnaire; Questionnaire to assess knowledge of eating disorders; Sociocultural Attitudes Towards Appearance Questionnaire; Multidimensional Self-Concept Scale; Patient Health Questionnaire-9; Generalized Anxiety Disorder Scale. Data collection: begin 02/2012, end 07/2014

The inclusion of female and male adolescents at all levels of eating disorder risk without prior screening, the effort to include as many schools as possible, the focus on the reduction of risk for eating disorders and the promotion of protective factors as well as the inclusion of input from community stakeholders (Center of the Prevention of Addiction, CPA) were features which formed the universal approach of the present program [37]. The program comprised three lessons which were delivered over two consecutive weeks (270 min dose in total). Females and males engaged together in the present program. Figure 1 provides an overview of the content and course of the intervention.

During the first 90-min-lesson students defined and critically discussed Western beauty ideals for female and male adolescents. This intervention was followed by an individual reflection of own investments to follow beauty ideals and a media literacy intervention focusing on the influence of social media and advertisements on the individual pressure to suit Western beauty ideals and how to cope with this pressure. The first lesson ended with an interactive intervention to help adolescents to cope with pressure from peers and family. One week later, female and male students past the second 90-min-lesson which comprised an interactive nine station parcour which addressed risk factors for eating disorders, protective factors and healthy nutrition (see Fig. 1). At each station, participants were provided with gender-adapted materials, e.g. different instructions for female and male participants. Adolescents passed each station in couples to allow for focused and open discussions. The third lesson directly followed the second lesson and was used to increase students’ awareness towards local and online help-seeking in case of mental health issues as well as to develop coping strategies for the whole class if a class mate suffers from an eating disorder. A detailed description of the prevention materials and the dissonance-based approach of the prevention program is provided in the study protocol [25]. The prevention materials were piloted in one school in January 2012. As a result, instructions for some interventions were revised. A prevention manual was then created to standardize the oral and written information during implementation. The intervention was conducted between February 2012 and January 2014.

### Statistical methods

The sample size was calculated for the prespecified contrast in the primary outcome, i.e., the adjusted mean difference in the ChEDE-Q change scores between the intervention and control groups from baseline to follow-up [38]. Change scores were applied to handle baseline differences between intervention and control group.

For a two-sided test with a type I error rate of 5%, power of 80% and an effect size (*ES*) of *Cohen’s d* = 0.30 [12], two samples with 176 participants each (*N* = 352) are needed to reach statistical significance. With an expected intracluster correlation of .05 and an average cluster size of 65 participants, the design effect is 4.6, which increased the required sample size to 739 participants per group (*N* = 1478). With an additional 25% loss to follow-up, a total of 1848 participants needed to be recruited.

We assessed baseline descriptive statistics for all randomly assigned participants by treatment allocation. The primary outcome hypothesis was tested using a random-effects analysis of covariance (ANCOVA; [39]) with school as a random factor; with group (intervention vs. control), sex (male vs. female) and grade (8th vs. 11th) as fixed factors; and with baseline eating disorder risk as a continuous covariate using the complete case population at follow-up (although parental educational level appeared in the study protocol, it was not included as a fixed factor because 31% of the participants did not know their parents’ educational level). The intracluster correlation coefficient, a measure of the heterogeneity of the average responses between schools, was calculated and tested based on the random terms of the model.

In a secondary analysis, we conducted an analogous random-effects repeated-measures ANCOVA at the post-intervention and follow-up points for each prespecified secondary outcome while allowing for heteroscedasticity and autocorrelation. In each case, the time of assessment (post, follow-up) was included as a repeated measure, and the significance of the time*group interaction was examined. These secondary analyses were adjusted for the influence of baseline levels from the respective secondary outcome. Preliminary analyses revealed significant differences between students who did not complete follow-up assessments and students who were “completers”. In addition to the control variables documented in the study protocol, BMI and baseline eating disorder risk were included as continuous control variables in all analyses.

As post hoc subgroup analyses of the primary endpoint, a dummy variable including time (post, follow-up) and grade (8th vs. 11th) was coded, and the group*(time/grade) and sex*(time/grade) interactions were analyzed.

An additional secondary analysis aimed to improve the understanding of the interdependencies between the primary and secondary outcomes. Therefore, when a significant effect in the secondary outcomes was observed, the SPSS markro PROCESS [40] was used to analyze a potential mediating effect on the primary endpoint. These secondary analyses were first conducted in the complete case sample and then repeated in the subsamples of participants from grade 8 and 11, respectively. All other analyses were first performed on completers with subsequent sensitivity analyses in the intention-to-treat (ITT) population (participants with a baseline ChEDE-Q score). The multiple-imputation algorithm provided by SPSS was used to address missing values [41].

Two-tailed *p*-values <.05 were considered significant. Adjusted means with 95% confidence intervals (CIs) are represented in figures and tables. The data were analyzed using IBM SPSS Statistics version 21.0. and SAS Version 9.4 (SAS Institute Inc., Cary, NC, USA) was used for randomization.

## Results

**Fig. 2:**
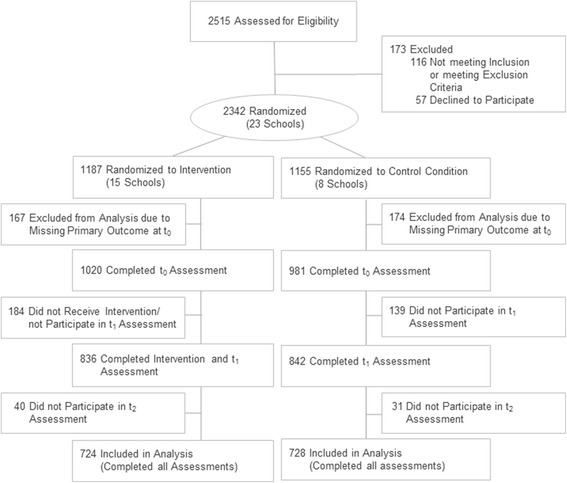
Flow diagram of the progress through the phases of the study

A total of 23 schools agreed to participate in the study. Of those, 15 schools (*n* = 1187 students) were randomly assigned to the intervention condition and eight schools (*n* = 1155 students) to the control condition (*N* = 2342; Fig. 2). The participation rates for the intervention group were 88% at post-intervention and 86% at follow-up as well as 85% and 89% for the control group. In grade 11, frequencies of male and female students who did not complete follow-up assessments (“non-completers”) did not significantly differ (30.3% vs.26.7%; *X* ^2^ = 1.245, *p* = .26). However, in grade 8, significantly more male students were “non-completers” compared to female students (30.4% vs. 23.8%; *X* ^2^ = 6.829, *p* = .01).

Intervention and control group participants were well balanced with respect to all sociodemographic variables (Table 1).

**Table 1:** Baseline characteristics of the complete case sample separated by group (intervention and control; *N* = 1452) and comparison of completers vs. non-completers (*N* = 2001)

Characteristic	Intervention *n* = 724	Control *n* = 728	*p*	Completer *n* = 1452	Non-Completer *n* = 549	*p*
Sex			.38			.005
Female – n (%)	393 (54.3)	415 (57.0)		808 (55.6)	268 (48.8)	
Age^a^ - M (SE)	14.4 (0.30)	14.8 (0.35)	.45	14.6 (0.22)	14.9 (0.23)	.002
BMI^a^ - M (SE)	19.8 (0.24)	20.0 (0.28)	.61	19.9 (0.17)	20.2 (0.19)	.003
Grade			.62			.55
8th grade – n (%)	491 (67.8)	414 (56.9)		905 (62.3)	333 (60.7)	
Migration background^a^			.34			.25
No migration background – n (%)	487 (68.1)	513 (72.1)		1000 (69.6)	347 (64.6)	
One parent – n (%)	118 (16.5)	86 (11.9)		204 (14.2)	86 (16.0)	
One parent and participant or both parents – n (%)	92 (12.9)	88 (12.2)		180 (12.5)	82 (15.3)	
Both parents and participant – n (%)	18 (2.5)	34 (4.7)		52 (3.6)	22 (4.1)	
Parents educational level^a^			.87			.55
Low both parents – n (%)	78 (15.8)	92 (18.2)		170 (17.0)	88 (23.6)	
High one parent – n (%)	94 (19.1)	124 (24.5)		218 (21.8)	79 (21.2)	
High both parents – n (%)	321 (65.1)	290 (57.3)		611 (61.2)	206 (55.2)	

*Note*: ^a^Variable contains missing values. Percentages may be calculated using different denominators due to missing values. Non-completers are students who did not complete the follow-up assessments; completers are students who completed all follow-up assessments

In contrast to our primary hypothesis, we did not observe a significantly greater decrease in eating disorder pathology in the intervention group compared to the control group at follow-up (Table 2). However, in line with our secondary hypotheses, the intervention group displayed a significantly greater increase in eating disorder related knowledge than the control group after the intervention and at follow-up. Moreover, compared with the control group, the intervention group showed significantly greater improvements in anxiety symptoms and a greater increase in experienced pressure due to the internalization of Western beauty ideals directly after the intervention but not at follow-up. The intervention group did not exhibit superior change scores regarding self-concept and depression symptoms at either time of assessment (Table 2).

**Table 2:** Baseline and follow-up assessment outcomes based on least-squares mean estimates of a mixed-model analysis within the complete case sample (*n* = 724 intervention; *n* = 728 control participants)

	Intervention group (*n* = 724)			Control group (*n* = 728)			Group^a^Time Inter-action *p*	Between-group differences		
	Raw mean (SD)	Change to baseline		Raw mean (SD)	Change to baseline			Adjusted mean difference (95%CI)		
Outcome variables		Adjusted mean (95% CI)	*p*		Adjusted mean (95% CI)	*p*			*p*	*ES*
Primary outcome^a^										
ChEDE										
Baseline	0.82 (1.08)			0.84 (1.01)						
6 months follow-up	0.79 (1.07)	−0.06 (−0.17; 0.04)	.22	0.79 (1.07)	−0.05 (−0.16; 0.07)	0.38		−0.01 (−0.17; 0.14)	.85	
Secondary outcomes^b^										
ChEDE										
Baseline	0.82 (1.08)			0.84 (1.01)						
Post-intervention	0.81 (1.10)	−0.04 (−0.12; 0.05)	.37	0.82 (1.02)	−0.07 (−0.16; 0.02)	0.11		0.03 (−0.08; 0.15)	.54	
6 months follow-up	0.79 (1.07)	−0.05 (−0.13; 0.03)	.21	0.86 (1.02)	−0.03 (−0.12; 0.06)	0.43	0.118	−0.02 (−0.14; 0.10)	.77	
Knowledge										
Baseline	8.72 (2.93)			9.13 (2.68)						
Post-intervention	11.91 (3.61)	3.12 (2.64; 3.60)	<.01	9.21 (2.89)	0.12 (−0.43; 0.67)	.65		3.00 (2.27; 3.72)	<.01	1.06
6 months follow-up	10.35 (3.37)	1.56 (1.08; 2.04)	<.01	9.65 (3.04)	0.56 (0.01; 1.10)	.05	<.001	1.00 (0.01; 1.73)	.01	0.40
SATAQ Internalization										
Baseline	11.50 (5.96)			11.80 (5.75)						
Post-intervention	11.95 (6.14)	0.40 (−0.03; 0.82)	.07	11.83 (5.74)	−0.01 (−0.47; 0.45)	.97		0.40 (−0.22; 1.03)	.18	
6 months follow-up	11.61 (5.93)	0.07 (−0.36; 0.49)	.75	12.04 (5.89)	0.20 (−0.26; 0.65)	.37	0.02	−0.13 (−0.75; 0.49)	.66	
SATAQ Awareness										
Baseline	13.42 (4.52)			13.89 (4.57)						
Post-intervention	13.83 (5.28)	0.35 (−0.13; 0.82)	.14	13.89 (4.73)	0.08 (−0.43; 0.59)	.74		0.27 (−0.43; 0.96)	.42	
6 months follow-up	13.51 (5.01)	0.03 (−0.44; 0.50)	.90	14.12 (4.73)	0.30 (−0.21; 0.81)	.23	0.02	−0.27 (−0.96; 0.42)	.42	
SATAQ Pressure										
Baseline	8.96 (4.74)			9.13 (4.52)						
Post-intervention	10.16 (5.00)	1.20 (0.94; 1.46)	<.01	9.67 (4.78)	0.54 (0.29; 0.79)	<0.001		0.66 (0.31; 1.02)	<.01	0.10
6 months follow-up	9.66 (4.86)	0.70 (0.44; 0.96)	<.01	10.08 (4.95)	0.95 (0.69; 1.20)	<0.001	<.001	−0.25 (−0.61; 0.11)	.17	
MSCS General										
Baseline	111.56 (19.28)			111.13 (18.08)						
Post-intervention	111.83 (19.95)	0.52 (−1.24; 2.28)	.54	112.96 (18.70)	2.43 (0.51; 4.35)	.02		−1.91 (−4.50; −0.68)	.13	
6 months follow-up	112.88 (20.85)	1.57 (−0.19; 3.33)	.08	113.24 (19.28)	2.71 (0.79; 4.64)	.01	0.36	−1.14 (−3.74; 1.45)	.35	
MSCS Body Related										
Baseline	46.54 (12.04)			47.45 (11.57)						
Post-intervention	46.07 (11.90)	−0.48 (−1.28; 0.32)	.23	47.37 (11.81)	0.56 (−0.26; 1.37)	.17		−1.04 (−2.17; 0.09)	.07	
6 months follow-up	47.27 (12.39)	0.72 (−0.08; 1.51)	.08	48.25 (11.99)	1.44 (0.62; 2.25)	<.01	0.53	−0.72 (−1.85; 0.41)	.20	
PHQ-9										
Baseline	5.41 (4.32)			5.39 (4.26)						
Post-intervention	5.16 (4.61)	−0.16 (−0.52; 0.19)	.34	5.20 (3.94)	−0.25 (−0.64; 0.14)	.18		0.09 (−0.43; 0.61) 0.07 (−0.45; 0.59)	.73 .78	
6 months follow-up	5.28 (4.37)	−0.04 (−0.40; 0.31)	.80	5.34 (4.07)	−0.11 (−0.50; 0.27)	.54	0.92			
GAD-7										
Baseline	3.90 (3.93)			3.94 (3.86)						
Post-intervention	3.21 (3.94)	−0.65 (−1.00; −0.29)	<.01	3.95 (3.97)	−0.01 (−0.40;0.38)	.95		−0.63 (−1.16; −0.10)	.02	0.22
6 months follow-up	3.62 (3.98)	−0.24 (−0.60; 0.12)	.18	3.92 (3.99)	−0.05 (−0.44; 0.35)	.80	0.01	−0.19 (−0.72; 0.34)	.45	

*Note*. *ChEDE-Q* Children’s Eating Disorder Examination Questionnaire, *PHQ-9* Depression Module of Patient Health Questionnaire, *GAD-7* Generalized Anxiety Scale, *SATAQ* Sociocultural Attitudes Towards Appearance Questionnaire, *MSCS* Multidimensional Self-Concept Scale; general, body related

^a^ Random-effects analysis of covariance

^b^ Random-effects repeated-measures analysis of covariance. Adjusted mean scores are controlled for BMI, baseline values, sex, grade and ChEDE-Q baseline score

**Fig. 3:**
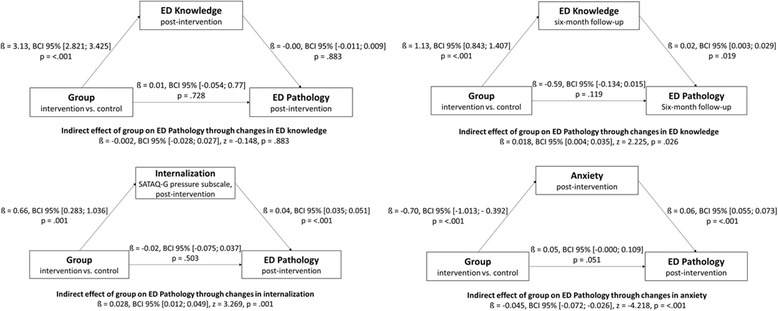
Mediation analyses in the complete case sample that examine indirect effects between group allocation and changes in eating disorder pathology. *Note.* ED = eating disorder; BCI 95% = 95% bootstrap confidence intervals; Internalization = pressure subscale of the Sociocultural Attitudes Towards Appearance Questionnaire

Taking into account the secondary outcomes and times of assessment in which a significant effect was observed, mediation models in the complete case sample revealed that changes in eating disorder knowledge at follow-up but not at post-intervention as well as changes in anxiety and pressure to suit Western beauty ideals at post-intervention partially mediated the relation between group allocation and changes in eating disorder pathology (Fig. 3). However, different indirect effects were observed in grades 8 and 11. In grade 8, changes in pressure to suit Western beauty ideals at post-intervention (*ß* = 0.033, *BCI* 95% [0.013; 0.061], *z* = 3.070, *p* = .002) and changes in eating disorder knowledge (*ß* = 0.024, *BCI* 95% [0.007; 0.049], *z* = 2.240, *p* = .025) mediated the relation between group allocation and changes in eating disorder pathology. In grade 11, changes in anxiety at post-intervention (*ß* = −0.061, *BCI* 95% [−0.129; −0.026], *z* = −3.826, *p* = <.001) mediated the relation between group allocation and changes in eating disorder pathology.

**Fig. 4:**
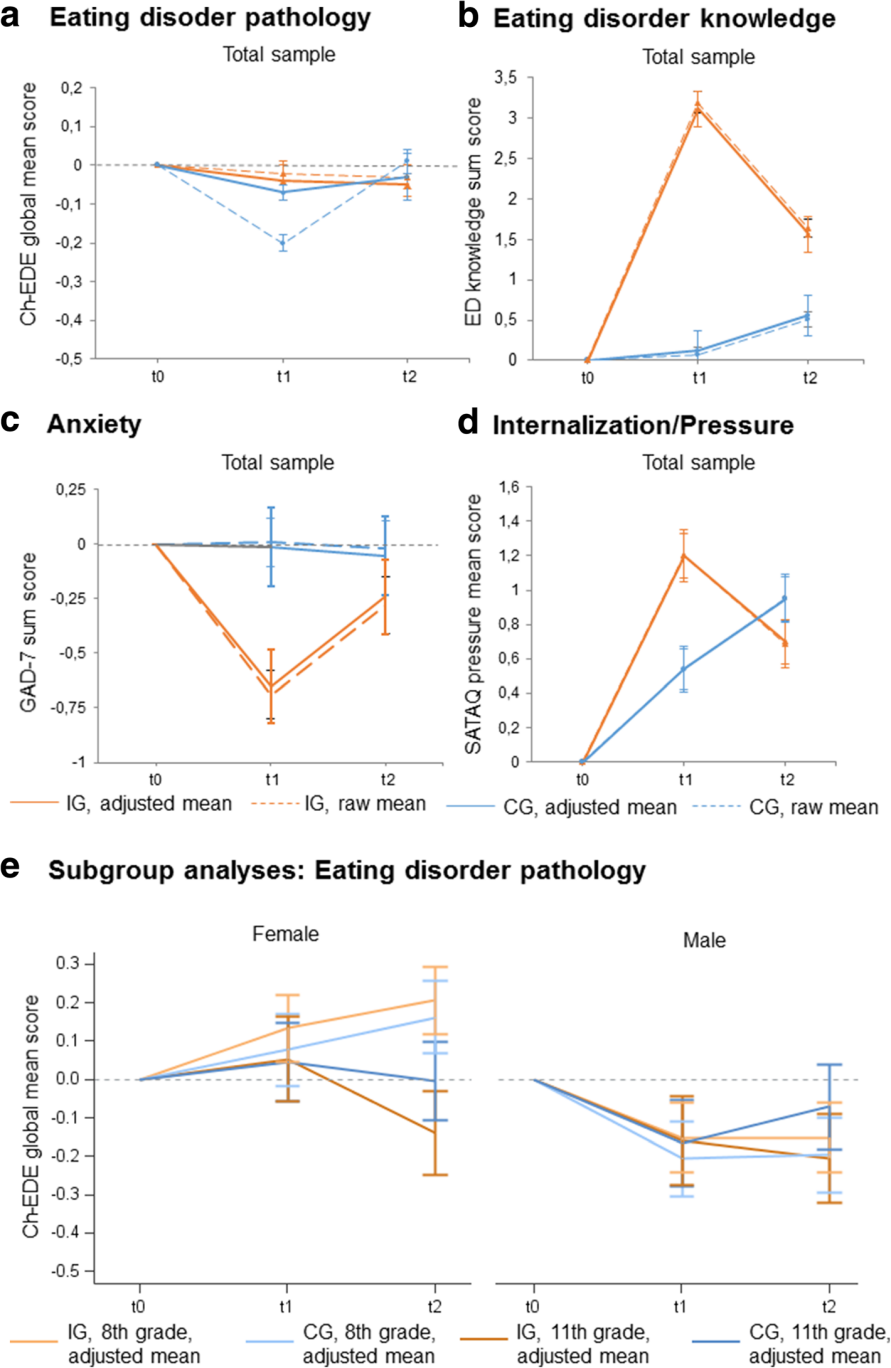
Mean changes from baseline to post-intervention and six-month follow-up for the primary and selected secondary outcomes. *Note*. t_0_ = Baseline assessment, t_1_ = post-intervention, t_2_ = six months post-intervention. **a** Children’s Eating Disorder Examination Questionnaire global mean score (primary outcome, scale range 0–6, negative change scores are desired); **b** Eating disorder knowledge sum score (secondary outcome, scale range 0–20, positive change scores are desired); **c** Generalized Anxiety Disorder Scale global sum score (secondary outcome, scale range 0–21, negative change scores are desired); **d** Sociocultural Attitudes Towards Appearance Questionnaire, pressure subscale sum score (secondary outcome, scale range 5–25, negative change scores are desired); **a-d** means are shown as raw means and adjusted for sex, grade, body mass index, baseline Ch-EDE-Q score; **e** Children’s Eating Disorder Examination Questionnaire global mean score (post hoc analyses of primary outcome regarding significant interactions between group, grade and time as well as between sex, grade and time, scale range 0–6, negative change scores are desired), means adjusted for sex, grade, body mass index, baseline Ch-EDE-Q score. CG = control group, IG = intervention group; * *p* < 0.05, **p < 0.01, ***p < 0.001

Accordingly, we observed a significant interaction between participants’ group allocation, time of assessment and grade in the post hoc analyses (complete case sample *p* = .01, ITT sample *p* = .049). As Fig. 4 illustrates, 11th grade female and male participants in the intervention group exhibited a lower eating disorder pathology than the participants in the control group at follow-up which was partially in line with our hypotheses, while 8th grade participants showed the opposite trends (interaction *p* = .012).

A second significant interaction between participants’ sex, time of assessment and grade (complete case sample *p* = <.001, ITT sample *p* = <.001) indicated that in both groups and at both time points, 8th grade females exhibited an increased eating disorder pathology, whereas in 8th grade males, the risk decreased directly after the intervention and subsequently remained stable (interaction *p =* < .001).

ITT analyses confirmed the complete case results (see Additional file 2: Table S1 available online).

## Discussion

This cluster-randomized controlled trial was designed to evaluate the impact of a three lesson prevention program on eating disorder pathology for female and male adolescents. Regarding the primary endpoint, we did not find the expected difference in the change in eating disorder pathology between the intervention and control groups at six months follow-up. Regarding the secondary endpoints, adolescents who participated in the intervention as expected exhibited a persistent and larger increase in knowledge at both times of assessment compared to the control group which is in line with a previous meta-analytic review that observed higher effect sizes for outcomes related to eating disorder knowledge [12, 42]. Additionally, adolescents who participated in the intervention displayed a greater decrease in anxiety post-intervention compared with the control group. In contrast to our expectations, levels of depression and self-esteem were not positively influenced by the intervention. Moreover, the intervention group showed a greater increase in experienced pressure due to the internalization of Western beauty ideals directly after the intervention. This increase might be attributed to the increased awareness towards the presence of and pressure from Western beauty ideals and interpreted as a consequence of cognitive dissonance. Taken together, the present universal prevention program for eating disorders resulted in some positive effects but did not decrease eating disorder pathology. One might argue that this lack of an effect on the primary endpoint might be attributed to the low dosage of the present prevention program. It was the aim of the present project to develop a prevention program with a high likelihood of a long-term implementation in schools. Also, the effects observed in the present study were comparable to those observed in universal prevention programs with a higher dosage (for an overview see [14]). Thus, the efficacy of universal prevention programs for eating disorders can not only be attributed to their quantity.

Eating disorder pathology scores of male and female adolescents developed differently in the intervention and control groups as well as in grades 8 and 11. In grade 8 females, an increase in Ch-EDE scores from baseline to post-intervention and six months later was observable in both the intervention and control group (Fig. 4). No such increase in eating disorder pathology could be observed in grade 8 male adolescents from both groups. In contrast to grade 8 adolescents, the decrease in Ch-EDE-Q scores in 11th grade adolescents indicated that this subgroup seem to have benefited from the prevention program with regard to their eating disorder pathology. One explanation for the varying effects of the present program might be that the mechanisms of efficacy are influenced by the age of delivery. In this context, the results of the mediation analyses revealed that in grade 8 changes in eating disorder knowledge significantly mediated the relation between group allocation and changes in eating disorder psychopathology at follow-up. A positive regression coefficient indicated that a knowledge increase was associated with an increase in eating disorder psychopathology. However, a confidence interval very close to zero pointed in the direction that this effect might not be clinically relevant. A similar picture occurred with regard to perceived pressure to suit Western beauty ideals where a significantly mediating effect was observed in grade 8 but not in grade 11. In this age group no such significant mediating effects of eating disorder knowledge or perceived pressure to suit Western beauty ideals could be observed. When one compares the content of the present eating disorder prevention program with those developed by Wilksch and colleagues [16] it is noteworthy that the prevention program with the most favorable outcome (Media Smart) focused heavily on media internalization, media transported stereotypes and pressure from media whereas the programs with iatrogenic effects focused either on a healthy lifestyle (Life Smart) or included interventions addressing appearance related issues (HELLP). The present program comprised interventions comparable to all three programs. Thus, with regard to participants from grade 8 it seems reasonable for future prevention programs to conduct dismantling investigations to a) examine whether addressing media internalization is truly the most promising approach for universal prevention programs and b) whether it is favorable to focus on a small number of adressed risk factors to ensure that the content is elaborately learned rather than targeting multiple risk factors. On a meta-level our results might provide further evidence that eating disorder knowledge increase should not be an intervention goal and part of primary or secondary endpoints in universal prevention programs that address young adolescents.

The specific effect of the intervention on 11th grade participants may be regarded as significant because an increase in eating disorder pathology has to be expected particularly in late adolescent females [3] and late adolescence is the peak age of onset for bulimia nervosa and binge eating disorder [10]. Participants from grade 11 differed from grade 8 participants in that in grade 11 anxiety mediated the relation between group allocation and changes in eating disorder psychopathology at post-intervention. This result is in line with previous evidence that identified anxiety as a risk factor for eating disorder symptomatology in female adolescents [43]. Furthermore, in this age group a knowledge increase did not result in an iatrogenic effect and might contribute to greater symptom awareness during later adolescence and facilitated help-seeking behavior in affected individuals and their peers [44].

Previous studies have shown that prevention programs for adolescents in the eating disorder field tend to be more effective when delivered to females or when solely addressed to either females or males who already show an elevated risk [12, 45]. In our view, the applicability of universal prevention programs to entire classes involving both females and males through gender-adapted materials appears to be important. Such an approach can help to increase awareness of eating disorder risk factors, such as peer pressure, thereby decreasing the stigmatization of mental illnesses and improving pro-social behavior in classes for the most relevant peer group during adolescence [9]. Furthermore, a long term implementation might be facilitated if schools do not need to provide alternative activities for males.

The strengths of our study include a sound study design with cluster randomization and follow-up assessment as well as the high number of participating schools and adolescents. The high practicability of the gender-adapted materials, the brevity of the intervention, and the close cooperation with an established stakeholder of school-based prevention are further strengths of this research.

The results of the study should be interpreted in light of the following limitations. First, individual randomization seemed neither feasible nor appropriate to evaluate the present program [46]. A cluster randomization of classes would have been more methodologically rigorous given that students within the same school might be more similar than compared with students from other schools. However, participating schools refused a randomization of classes. Cluster randomization with schools as the unit of randomization facilitated the implementation of the intervention and prevented control participants from being unintentionally affected by the intervention. Moreover, we considered it more suitable for students to be in their current peer group, as the present prevention materials comprised several interactive interventions, i.e., group discussions. Second, concealing the allocation of treatment from schools, students or parents was not feasible. This non-blinded allocation might have resulted in an expectancy effect. Allocation to the control group for example might have reduced schools’ commitment to take part in the study, it might have influenced students’ responses to the outcome measures and parents’ behavior towards their children. With the aim to overcome this limitation and to ensure schools’, students’ and parents’ commitment, the prevention program was offered to participants in the control group after the follow-up assessment. Third, we observed a low response rate of schools who were willing to participate in the present study together with a high attrition rate of schools who consented to participate. This might have decreased the representativeness of our sample in that only particularly motivated schools agreed to participate. It has to be kept in mind, however, that during the implementation of the study important changes in the Hamburgian school system occurred. Many schools which might have been otherwise motivated to participate in the present study were occupied with restructuring activities. Of the participating schools, only one had gone through restricting activities prior to participation. On a more general level, the low response rate of schools highlights the great need to increase the motivation of schools to implement prevention programs. Fourth, the self-report measures were answered during classes; hence, social desirability bias may have affected the completion of these measures. We attempted to limit this potential bias through the attendance of research assistants who ensured that the questionnaires were completed discreetly and quietly.

## Conclusions

In summary, the present prevention program can be recommended to older adolescents where it resulted in increased knowledge on eating disorders and a decrease in eating disorder pathology. We do believe that knowledge of mental illnesses should be an integral part of education and that prevention programs might also contribute to destigmatization and early detection of mental diseases. Future studies should focus on the evaluation of the present program in grade 11 adolescents and examine the possibility of a teacher- or peer-based delivery of the intervention.

## Additional files

Additional file 1: Study protocol. (PDF 402 kb)

Additional file 2: Table S1. Baseline and follow-up assessment outcomes based on least-squares mean estimates of a mixed-model analysis within the intention-to-treat sample (*n* = 1020 intervention participants; *n* = 981 control participants). (DOCX 21 kb)

## Abbreviations

BMI: Body-Mass-Index
ChEDE-Q: Eating Disorder Examination Questionnaire – children’s version
CPA: Centre for the Prevention of Addiction
GAD-7: Generalized Anxiety Disorder Scale
MSCS: Multidimensional Self-concept Scale
PHQ-9: Patient Health Questionnaire – Depression Module
SATAQ: Social Attitudes Towards Appearance Questionnaire
