# Supplementary material for: Efficacy of a prevention program for eating disorders in schools: a cluster-randomized controlled trial
Source: BMC Psychiatry. 2017 Aug 11;17:293. doi: 10.1186/s12888-017-1454-4 (PMC5553667; doi:10.1186/s12888-017-1454-4)
Supplement: Supplementary file 1 — Study protocol. (PDF 402 kb) [file 12888_2017_1454_MOESM1_ESM.pdf]

Weigel et al. BMC Psychiatry (2015) 15:21
DOI 10.1186/s12888-015-0405-1

BMC Psychiatry

STUDY PROTOCOL Open Access

Preventing eating disorders with an interactive gender-adapted intervention program in schools: Study protocol of a randomized controlled trial

Angelika Weigel[1*†], Antje Gumz[1,4†], Natalie Uhlenbusch[1], Karl Wegscheider[2], Georg Romer[3] and Bernd Löwe[1]

Abstract

Background: There are a high number of adolescents who are at risk of developing an eating disorder. There is, therefore, a strong need to implement prevention programs aimed at reducing the incidence of eating disorders at this critical age. Among other factors, successful prevention programs have been shown to be interactive, carried out by professionals, focused on educational as well as psychosocial elements and have taken risk factors as well as resources into account. The objective of this study protocol is to present the design of a new prevention program for eating disorders in schools.

Methods/Design: The gender-adapted prevention program extends over six school hours. It contains interactive and educational elements about eating disorders and their treatment. Participants pass through different exercises and reflect on the influences of the media, self-esteem, body perception and individual resources. A cluster-randomized controlled trial is chosen to evaluate the program. Based on an estimated effect size of d = 0.3 a total of 1848 participants are enrolled in the study. Eating disorder risk, internalization of Western beauty ideals, body dissatisfaction, self-concept as well as anxiety and symptoms of depression are measured before and immediately after the intervention as well as at a six-month follow-up. In addition, the intervention group evaluates the different components of the program.

Discussion: The study intends to test the practicability and efficacy of an interactive, gender-adapted ED prevention program in schools. Moreover, it will provide valuable information about the occurrence of eating disorder risk factors in school-aged children.

Trial registration: ISRCTN97989348; Registered 19 December 2012.

Keywords: Prevention, Eating disorders, Adolescents, Mental health, Schools

Background

The Diagnostic and Statistical Manual of Mental Disorders describes different types of eating disorders (EDs) (DSM-V) [1]: Anorexia nervosa (AN), Bulimia nervosa (BN), Binge Eating Disorder (BED) and Eating Disorders Not Otherwise Specified (EDNOS). What is common to all disorders is a definite disturbance of eating habits or weight control behaviors and patients often transition between fulfilling the criteria for different diagnoses [2]. EDs occur remarkably less frequently than other mental disorders. The 12-month prevalence in the general population of any ED is 0.9% [3]. AN was found to be the most frequent with a 12-month prevalence of 0.7%, followed by a prevalence of 0.2% for BN and 0.1% for BED [3]. Patients suffering from an ED often also suffer from other psychiatric disorders [4] with mood and anxiety disorders being the most common comorbidities [5]. In addition, EDs are often accompanied by severe physical consequences [2]. EDs occur significantly less frequently in males than in females with a proportion of 10% of all cases in AN and BN and 30% in BED [6]. Despite, affected males suffer from similar psychosocial morbidity [7]. As well as the individual burden, the treatment of EDs is also associated with high health care system costs [8].

* Correspondence: a.weigel@uke.de
†Equal contributors
[1]Department of Psychosomatic Medicine and Psychotherapy, University Medical Center Hamburg-Eppendorf & Schön Klinik Hamburg Eilbek, Martinistraße 52, 20246 Hamburg, Germany
Full list of author information is available at the end of the article

BioMed Central

© 2015 Weigel et al.; licensee BioMed Central. This is an Open Access article distributed under the terms of the Creative Commons Attribution License (http://creativecommons.org/licenses/by/4.0), which permits unrestricted use, distribution, and reproduction in any medium, provided the original work is properly credited. The Creative Commons Public Domain Dedication waiver (http://creativecommons.org/publicdomain/zero/1.0/) applies to the data made available in this article, unless otherwise stated.

A multifactorial model of the etiology of EDs is generally accepted in the literature [9] and particular risk factors have been identified. Table 1 provides an overview of well-established risk factors for school-aged children and adolescents (for more detailed information see e.g. [10]).

Children and adolescents are at particular risk of developing EDs. In AN, for example, there is evidence of two peaks of onset, one before 15–16, the other after 18 years [18]. BN and BED occur later with an average age of onset of approximately 20 years in BN and BED [10]. In addition, the beginning of subclinical levels of eating pathology and disordered eating behavior has been observed in this age range. The German Federal Center for Health Education (BZgA) has reported that more than half of German 13- to 14-year-olds would like to be thinner [19]. Another study with 14- to 18-year-olds showed that 42% of boys and 53% of girls preferred a thin body ideal, 18% of the boys and 19% of the girls were currently trying to lose weight and 17% of the children with a normal weight felt too fat [20]. Moreover, 28.9% of the 11- to 17-year-old girls and 15.2% of the boys showed pathological eating behavior [21]. Such results suggest, therefore, that school age might be an ideal time to implement preventive interventions.

The media might play an important role in the internalization of Western beauty ideals. It is estimated that only one out of every 40.000 women meets the height, weight and body shape criteria of professional models [22], which can lead to body dissatisfaction. This body dissatisfaction is often accompanied by depressive states [23,24] as well as low self-esteem [25] and makes the development of an ED more likely [15].

Schools offer a convenient framework for the implementation of preventive interventions. Children are exposed to social pressure at school which is a significant factor in the development of a negative body image as well as weight concerns in girls and boys alike [26]. Another advantage of schools as the setting for prevention interventions is the possibility to reach a large number of people at the same time. Many previous interventions have only included small samples (for an overview see [27]). Since prevention should benefit individuals as well improve collective health [28], programs should reach as many individuals as possible.

Table 1 Risk factors for Eating Disorders with good empirical support

Risk factors	Study
**Individual risk factors**	
Female sex	[2,11-13]
Adolescence and childhood	[2,11-13]
Low self-esteem	[11-13]
Perfectionism	[14,15]
Negative emotionality	[11-15]
Body dissatisfaction	[15,16]
**Psychosocial risk factors**	
Internalization of Western beauty ideals	[14,15,17]
Adverse life events (e.g. sexual abuse)	[2,11-13]

It has been shown that prevention programs developed and conducted in different countries are able to decrease individual ED risk and reduce the occurrence of EDs. In a brief, 8-week, internet-based, cognitive-behavioral intervention in Californian college-age women who were at risk of developing EDs, weight and shape concerns were significantly reduced [29]. Two other intervention programs carried out in the United States for high-school girls with an elevated ED-risk resulted in a significant reduction of bulimic pathology as well as thin-ideal internalization and negative affectivity [30]. On a German national level, Berger, Strauss and colleagues developed and evaluated three different prevention programs in schools. After finishing the programs, the majority of children displayed less alarming eating behavior, greater appreciation of their bodies, increased knowledge about healthy nutrition and EDs as well as raised self-esteem [31-33]. It is important to note, however, that these interventions are time intensive and were evaluated in close cooperation with selected schools.

Taken together there is evidence that ED risk might be positively influenced by preventive interventions. Research has shown that successful ED prevention programs are interactive, selective for risk groups, offered only to females, offered to participants aged over 15 and delivered by professionals [27,34]. However, the young age of onset in AN makes prevention before the age of 15 necessary. In addition, other studies confirmed the “Life-skills” approach [35] which focuses on the improvement of competences in stress- and conflict management as well as strengthening self-esteem and empathy. Preventive interventions need to be able to be integrated in school schedules and address both female and male pupils to reach a broader school population. At the same time, the reduction of risk factors as well as strengthening of existing resources and protective factors should also be included.

Objectives

The primary aim of this study is to prevent adolescents from developing an ED. To achieve this objective, a gender-adapted prevention program is carried out in schools. The prevention program is interactive and carried out by professionals and based on research regarding the prevention of EDs. It includes both educational and psychosocial elements and focuses on risk factors as well as resources. The program is carried out in close cooperation with the Center of the Prevention of Addiction (CPA) an

established and experienced stakeholder in the field of prevention. The CPA is an experienced local provider of ED specific prevention in schools from the State of Hamburg Institute for Teacher Training and School Development. With this close cooperation we hope to improve the successful implementation and sustainability of the program. An additional aim is to evaluate the prevention program and to do so we have designed a cluster-randomized controlled trial. In this design, the intervention group answers the same questionnaire-set one month before the intervention takes place, immediately afterwards and at a 6-month follow-up. The control group answers the same questionnaire-set at the same time intervals without attending the intervention program.

The study is part of the project psychenet: Hamburg network for mental health which is funded by the German Federal Ministry of Education and Research (BMBF). The purpose of the eleven psychenet sub-projects is to establish innovative care models which aim to improve the prevention, diagnosis and treatment of people with mental illnesses in the Hamburg metropolitan region [36]. Psychenet is coordinated by the Cluster Agency Healthcare Hamburg in collaboration with the University Medical Center Hamburg-Eppendorf. More than 80 scientific and medical institutions, counselling centers, the Senate and the Chamber of Commerce of the Free and Hanseatic City of Hamburg, companies, as well as patients' and relatives' associations have been a part of the network since it was initiated in 2011.

Sub-project IX (Health network anorexia and bulimia) focuses on the improvement of the effectiveness and efficiency of ED-specific care. The sub-project is divided into two different intervention arms. This article will provide an overview of the cluster-randomized controlled trial on the prevention of EDs. An overview of the second intervention arm (an intervention focusing on treatment initiation in patients with AN) is described elsewhere [37].

Methods

Study design

A cluster-randomized controlled trial was designed to evaluate the efficacy of the prevention program. To this end, global ED risk, and knowledge about EDs as well as values of different ED risk factors are measured one month before, immediately following, as well as six months after the intervention in both conditions of the trial.

Ethics approval

The study procedure was reviewed and approved by the ethics committee of the Psychotherapist Chamber of Hamburg (approved on the 26th July of 2011) as well as the Hamburg supervisory school authority.

Study procedures

Inclusion and exclusion criteria

Participants need to be adolescents attending school in the 8th or 11th grades (age 14 and 17 years). In addition, written informed consent is required to participate in the study and participants under the age of 16 years are required to provide additional informed consent from parents. Exclusion criteria are insufficient knowledge of the German language as well as not providing informed consent.

Recruitment

All secondary school principals within the Metropolitan area of Hamburg are informed about the study purpose and procedure by letters and emails. Participating schools are randomized into the study condition (intervention vs. control) according to the order of application. Randomization is carried out by an external institution (Department of Medical Biometry and Epidemiology, University Medical Center Hamburg-Eppendorf). It is important to note that we were prevented from randomizing according to classes as well as classes switching study condition (e.g. intervention vs. control at the beginning of a new school year) by the Hamburg supervisory school authority. Schools are, therefore, the unit of randomization and they stay in the same condition for the entire study duration.

After randomization school principals are informed to which study condition their school is allocated (intervention vs. control) as well as the detailed study procedure. School principals then nominate suitable classes and propose convenient time slots in the school year. Teachers of participating classes are provided with a detailed schedule of the study and are also given the chance to contact the research team at any time. A letter of consent with study information is distributed to students and their parents two weeks before the study is conducted. These letters contain a detailed description of the study procedure (especially the necessity of randomization). Classes randomized to the control group are given the opportunity to receive the intervention after the completion of the last questionnaire. Study participants have the possibility to contact a psychotherapist from the research team to seek professional mental health help throughout the whole study duration.

Modules of the intervention

The intervention comprises three 90 – minute units which are delivered over a two week period. The first unit occurs on its own while the second and third units occur straight after each other one week later. The aim of this sequence is to increase the engagement in the prevention program via between session homework. The intervention is administered by four members of the CPA as

well as four members of the research team, all of whom follow a detailed manual. All sessions are led by a one member of the CPA and one member of the research team with at least masters' level degree and a background in clinical psychology. The usual class teacher also attends the sessions. Monthly review meetings are held to ensure that the prevention manual is being implemented to a uniformly high standard through the duration of the study.

Unit 1

The first unit begins with brief introduction of the study team and an overview of the prevention program. The first intervention of this unit aims to interactively define the different Western beauty ideals for young females and males as well as the associated positive and negative stereotypes, perpetuating factors and the influence of the media. Therefore, participants are separated into two male and two female groups. One female and one male group each describe what they imagine an ideal young female or male would look like on a poster. Then the results are presented and critically discussed amongst the whole group. The second intervention involves participants rating "How important is it for you to follow beauty ideals?" (0 = not at all, 10 = absolutely) on a visual analogue scale and completing a short checklist of things young people could do to conform to beauty ideals (e.g. physical activity, special clothing, and cosmetics). Female and male participants anonymously mark their answers on the appropriate poster with glue dots. Differences between females and males are then remarked upon and the impact of beauty ideals on everyday life is critically discussed amongst the whole group. Both interventions aim to encourage individual self-reflection on the internalization of Western beauty ideals.

As part of the next intervention, participants watch a five-minute short-film of how models in advertisements are retouched. They are then encouraged to critically discuss how media transferred beauty ideals can be achieved using the things or activities indicated in the checklist if the media manipulates pictures used in advertisements and how the media benefits from the pursuit of beauty ideals.

The last intervention begins with a short recapitulation of the session (collective definition of beauty ideals for females and males, things done to conform to those ideals, role of the media) and then focuses on peer and family pressure. In this process participants provide examples of situations where they felt forced to change something about their body or clothing after comments from others and these examples are written on a large sheet of paper. Particular emphasis is made on the pressure to follow beauty ideals through Facebook and other social media. As homework, participants are asked to watch out for situations in everyday life where there is peer or family pressure and to consider solutions of how someone could react in these situations to positively withstand the pressure from others.

Unit 2

After an overview of the previous session, participants pass through an eleven station multi-methodological circuit in pairs. An evaluation sheet is handed out to each participant before the beginning of the circuit. This serves to monitor whether participants pass every station and how every station is evaluated (e.g. "Did you learn something relevant about yourself at this station? Did you learn something new at this station?"). Participants change stations every eight minutes and mark their answers on the evaluation sheet before moving onto the next station. At station 1, participants listen to a short mindfulness exercise to improve body perception on an mp3 player. Then they mark body parts they like, appraise as neutral or dislike on gender adapted figures. Station 2 aims to help participants identify their own resources. Different things which may help someone be self-confident are listed on a worksheet and participants are encouraged to find own examples. Participants then write the three most important resources to them on a small card. This card is then used later at station 3. There, participants find a wide choice of pictures showing positive activities one can do to cope with negative emotions. Things which the participants think might be helpful for them should be written on the backside of the small card. At station 4, participants take pictures of each other and critically discuss in pairs how they present themselves in social media. Station 5 comprises a puzzle about the physical consequences of different EDs. Participants find pictures of different body parts (e.g. liver, skin, hair) and need to find the corresponding written information. The solution is available at the end. At station 6, participants note the food they would eat on a typical school day and then discuss whether their nutrition is in line with the German Association of Nutrition's guidelines [38]. Participants can also get ideas on how nutrition can be improved by looking at a wide range of pictures of healthy food. Station 7 begins with a short audio sequence in which a boy visits his GP and the set-point theory is explained. Afterwards, participants complete a cloze text task. A solution word helps participants check whether all given answers are correct. At station 8, a counter-attitudinal role-play based on the homework from unit 1 is conducted. Each participant attempts to dissuade one of the group leaders from succumbing to peer or family pressure. At station 9, participants find gender-adapted descriptions of difficult situations as well as three respective coping strategies. The positive and negative consequences of each coping strategy are evaluated in pairs and participants are encouraged to choose a usable one. Station 10

involves a reciprocal interview on good reasons for and examples of healthy physical activity. Station 11 is the last of the circuit and includes an exercise to mindfully enjoy a piece of chocolate.

Unit 3

This unit begins after a regular school break. After re-entering the classroom participants are separated into three groups and each group is allocated one of three eating disorders (AN, BN or BED). A film is presented which shows three young people suffering from one of the EDs and group participants collect risk factors and symptoms described in the film about their allocated ED. Results are then presented to the whole class. Afterwards the groups are encouraged to reflect how they would react to and could support a person with an ED in their own class. Results are again presented to the class and the reduction of stigma of EDs is emphasized. After that, prior knowledge about treatment options for EDs and other mental illnesses is discussed and a group leader from the CPA provides the participants with information about ED-specific counselling-centers, psychotherapy as well as treatment options for different EDs. Participants are then able to pose any questions. The unit ends with a recapitulation of the whole prevention project and participants are encouraged to formulate their "lessons learned" during participation. After that, participants are asked to complete the post intervention questionnaire-set and are told that there would be a follow-up in six months. In addition to the post intervention questionnaire, a flyer is distributed in both study conditions (intervention and control) which includes the contact information of different treatment centers for EDs and other mental illnesses. Participants are invited to use information contained in the flyer if they feel that they themselves or a friend is in need of professional mental health help.

Variables and instruments

Participants of the intervention group complete a questionnaire at baseline, at the end of the intervention as well as six months post-intervention. Participants in the control group complete the questionnaire-set at equal intervals without receiving any intervention. The questionnaire-set contains socio-demographic information, standardized instruments to measure ED risk as well as ED risk factors, a knowledge test and an additional set of evaluative questions (only within the intervention condition of the study). Participants complete each questionnaire-set in class while trained research assistants with a background in clinical psychology answer any questions. No incentives for participation or feedback about individual test results are given to the participants.

Socio-demographic variables

Socio-demographic variables include school year (grade), Body Mass Index (BMI, weight in kilograms divided by height in meters squared), parental educational level, participants' current romantic relationship status, parental marital status, immigrant background as well as number of siblings, number of friends and amount of physical activity per week.

ED risk

ED risk is assessed by the German version of the Children's Eating Disorder Examination-Questionnaire (Ch-EDE-Q, [39]). The Ch-EDE-Q is the children's version of the Eating Disorder Examination Questionnaire (EDE-Q) for participants below the age of 16 years. The questionnaire assesses specific ED psychopathology on four subscales, which are restraint, eating concern, weight concern and shape concern. The calculation of subscale means as well as a total mean score is possible. The global score has a range from 0–132 points. Receiver Operating Characteristic (ROC) analysis showed an optimal trade-off between specificity and sensitivity at a mean global score of 2.3 (Se = .92, Sp = .86) with the EDE as diagnostic reference [40]. The psychometric properties of the Ch-EDE-Q have been shown to be satisfactory. It has adequate internal consistency in clinical samples (.65 ≤ Cronbach's α ≤ .88), excellent inter-rater reliability for the subscale scores as well as the global score ($.98 \leq r_{icc} \leq .99$) and acceptable retest reliability ($.24 \leq r_{tt} \leq .44$). Convergent and discriminant validity are also satisfactory [41].

Knowledge about ED

A 20-item questionnaire has been developed for the purposes of this study. It comprises questions regarding the content of the three units of the prevention program. Each question can be answered with "right"; "wrong" or "don't know". Every correctly answered question is given one point which is then added to give a sum-score. A maximum sum score of 20 points is, therefore, possible.

Internalization of Western beauty ideals

The internalization of Western beauty ideals transferred by the media is measured by the German version of the Sociocultural Attitudes Towards Appearance Questionnaire (SATAQ-G, [42]). The questionnaire assesses sociocultural influences on body image with three subscales: general internalization, awareness about media based beauty ideals as well as perceived pressure from the media to conform to beauty ideals. The questionnaire is available in gender-adapted versions, thereby taking the different body ideals of girls and boys into account. The instrument has been shown to be reliable with good internal consistency of the subscales (.75 ≤ Cronbach's α ≤ .89) and

acceptable convergent validity for the internalization and pressure subscales [43].

Body dissatisfaction
Figure drawings from the Kid's Eating Disorder Survey (KEDS, [44]) are used as a measure of body dissatisfaction. Eight figure drawings with increasing weight and size of body shape are presented for both sexes. Participants are asked to highlight their own real as well as their ideal body shape. The figure drawings are numbered in ascending order, "1" indicating the thinnest, and "8" indicating the largest body. Body dissatisfaction is calculated by subtracting the ideal from the real body shape. The KEDS has been shown to be a reliable instrument and the self-reported real body shape adequately represents current BMI [45].

Self-concept
Self-concept is assessed with the German version of the Multidimensional Self Concept Scale (MSWS, [46,47]). This instrument measures self-concept on six different subscales: emotional self-concept, security in social situations, handling criticism, performance-related self-concept, physical attractiveness and athletic self-concept. Psychometric qualities of the instrument are satisfactory with an internal consistency of $\alpha = .93$ and well demonstrated convergent, discriminant and construct validity [47].

Symptoms of depression
Symptoms of depression are assessed with the depression module of the Patient Health Questionnaire (PHQ-9, [48,49]). The self-assessment scale consists of nine items and it is able to establish provisional depressive disorder diagnoses and grade depressive symptom severity [50]. The questionnaire is reliable with an internal consistency of $\alpha = .89$ and a retest-reliability of $r_{tt} = .84$ [51].

Anxiety
Anxiety is assessed by the 7-item Generalized Anxiety Disorder Scale (GAD-7, [52]). There is evidence that the GAD-7 has good reliability as well as criterion, construct, factorial and procedural validity [53].

Sample size/power calculation
Participants are adolescents of grades 8 and 11 (age 14 and 17 years). Based on previous studies [46] an effect size of Cohen's $d = 0.3$ is expected. For a two-sided test with a type-I error rate of 5% and power of 80%, two samples with 176 participants each ($N = 352$) are needed to reach statistical significance. Given that cluster effects, expected inter-class correlations of 0.05 as well as an average cluster size of 65 participants need to be considered, the design factor is 4.2. Thereby at least 1478 participants need to be recruited. If an additional drop-out-rate of 25% is assumed then a total of 1848 participants are needed to be recruited.

Primary outcome
The primary outcome parameter is the ED risk status as metrically measured by the Ch-EDE-Q global mean score six months after participation in the prevention program. This would correspond Ch-EDE-Q global mean score differences between intervention and control group of 0.34 for female as well as 0.18 for male participants; based on the expected effect size of Cohen's $d = 0.3$ and standard deviations of 1.14 (females) and 0.61 (males) within comparable population based samples [54].

Secondary outcome
Knowledge about EDs, internalization of Western beauty ideals, body dissatisfaction, self-concept as well as symptoms of depression and anxiety are considered to be secondary outcomes.

Study hypotheses
It is hypothesized that the prevention program reduces the individual ED risk in participants of the intervention group compared to the control group. It is also expected that, participants of prevention program have increased knowledge about EDs, decreased body dissatisfaction and symptoms of depression as well as anxiety compared to the control group.

Handling of missing values
Participants with missing baseline ChEDE-Q global score will need to be excluded from the analysis. With the aim to include a maximum of participants in the analysis each questionnaire set will be labeled regarding group and sex. Information about these independent variables can, therefore, be derived if at least one questionnaire is answered. Also, information regarding the independent variables grade and type of school can be gathered from the documentation of the recruitment process.

Statistical analyses
In order to empirically test the hypotheses regarding differences between the intervention and control groups at the 6-month follow-up, multi-level analyses are conducted with school as a random factor as well as sex (male vs. female), grade (8th vs. 11th), parents' educational level (lower- both parents vs. higher – one parent vs. higher both parents) and group (intervention vs. control) as fixed factors and baseline ChEDE-Q scores as covariate. Two-sided tests with an alpha level of $p < .05$ and regression coefficients with 95% confidence intervals (CI) are calculated in all analyses. Analyses are first performed on completers

and then sensitivity analyses are conducted using imputed data.

Methods to reduce bias

Selection bias is minimized by the inclusion of a control group as well as the externally conducted cluster randomization of participating schools. The rate of participants or their parent who refrain from signing an informed consent (i.e. nonresponse rate) will be reported. The full recruitment process will be documented according to CONSORT reporting standards [54]. Surveys with high possibility of intentional misrepresentation (e.g. always choosing the one option of the likert scale) are considered as having dropped out. In addition, data is subjected to an internal validity check.

Study registration

Before starting, the trial was registered with an internet-based open access trial, controlled-trails.com (ISRCTN97989348; "Healthcare Network Anorexia and Bulimia nervosa"-campaign - Focal-project 1: Prevention trial at 20 secondary schools in Hamburg; Principal Investigator: Bernd Löwe; Registered 19 December 2012; Overall trial status: Ongoing; Recruitment status: No longer recruiting).

Discussion

This study is a cluster-randomized controlled trial evaluating a gender-adapted interactive ED prevention program carried out in schools. The prevention program includes educational elements about EDs and their treatment as well as interactive elements about potential risk factors such as the influence of the media, peer and family pressure, beauty ideals, self-concept, body perception as well as improving individual resources and their activation, coping strategies, social media, nutrition and healthy physical activity. It is hypothesized that the prevention program decreases individual ED risk in participants. It is also expected that the prevention program increases knowledge about EDs and reduces body dissatisfaction as well as symptoms of depression and anxiety. Moreover, it is hypothesized that these effects will be greater in the high-risk sample than the total sample.

Since EDs have a large negative impact on patients and their treatment is expensive and difficult, there is a definite need for effective prevention programs to reduce the number of new cases. Given the large number of participants, interactive nature of the program and it being conducted by professionals, the psychenet prevention program has the potential to have an important impact. By involving the CPA, an established and experienced stakeholder in the field of prevention, we hope we ensure successful implementation and sustainability of the program. Moreover, compared to many other intervention programs, the intervention presented here has a realistic and time efficient design to facilitate its long-term integration into school curricula. However, the brevity of the program may be disadvantageous in that the dose of the intervention may not be sufficient to observe the expected effects. We tried to overcome this disadvantage by implementing between session homework to encourage participants to extend the consideration of some of the program's messages beyond the classroom.

A further advantage of the prevention program is the adaption of material for both genders. Although prevention programs offered only to females have previously been shown to be more effective [27,34], a program including both sexes has several benefits: First of all, offers provided only for girls cannot be easily integrated into a typical school program. Moreover, in spite of the lower prevalence of EDs in males [6], there is strong evidence that EDs burden boys in a similar way to girls [7]. An intervention addressing both males and females may not only achieve an individual reduction of risk factors, the interaction between boys and girls might also be positively influenced.

In spite of the numerous advantages of the planned program and the pressing demand for prevention interventions, some limitations have to be considered: First, the implementation of the program by external professionals may encourage teachers to feel released of the responsibility to establish preventive interventions based on their own initiatives. After this program is first implemented, however, teachers may be included in the development of further preventive programs. Second, the program material is identical for both grades. It may be the case that the tasks are not challenging enough for the students in 11th grade whereas children in 8th grade may feel overburdened. Equivalent material in both groups is, however, necessary to properly evaluate the impact of the program. Questions to assess the perceived age appropriateness are included in the evaluation to help test this factor. Third, the repeated measurement of ED risk factors as well as symptoms within the control group might have a positive impact on participants in study condition. They might become more familiar with the topic of EDs and may feel encouraged to seek help themselves. If this is the case then the positive effects of the prevention program in the intervention group would be comparatively lower. The planned interventions will, however, make an important contribution to the prevention of EDs.

Abbreviations

EDs: Eating disorders; AN: Anorexia nervosa; BN: Bulimia nervosa; BED: Binge eating disorders; EDNOS: Eating disorders not otherwise specified; BZgA: Federal center for health education; CPA: Centre for the prevention of addiction; BMI: Body-mass-index.

Competing interests

The authors declare that they have no competing interests.

Authors' contributions

BL conceived and designed the study as well as obtained funding. AW and AG helped design the study and drafted the manuscript. NU helped draft the manuscript. All authors read and approved the final manuscript.

Acknowledgements

The study is one of eleven subprojects of a health service research project (psychenet) which is funded by the German Federal Ministry of Education and Research (BMBF; 01KQ1002B; Principal Investigator: BL). We would like to thank Dr. Alexandra Murray for her valuable comments on the manuscript as well as Amina Kuby for her translation of the ethnical and funding approval.

Author details

[1]Department of Psychosomatic Medicine and Psychotherapy, University Medical Center Hamburg-Eppendorf & Schön Klinik Hamburg Eilbek, Martinistraße 52, 20246 Hamburg, Germany. [2]Department of Medical Biometry and Epidemiology, University Medical Center Hamburg-Eppendorf, Martinistraße 52, 20246 Hamburg, Germany. [3]Clinic of Children and Adolescent Psychiatry, Psychosomatics and Psychotherapy, University Medical Center Münster, Schmeddingstr. 50, 48149 Münster, Germany. [4]Psychologische Hochschule Berlin, Am Köllnischen Park 2, 10179 Berlin, Germany.

Received: 4 December 2014 Accepted: 2 February 2015
Published online: 12 February 2015

References

1. American Psychiatric Association. The Diagnostic and Statistical Manual of Mental Disorders. Fifthth ed. Arlington VA: American Psychiatric Association; 2013.
2. Fairburn CG, Harrison PJ. Eating disorders. J Lancet. 2003;361(9355):407–16.
3. Jacobi F, Höfler M, Siegert J, Mack S, Gerschler A, Scholl L, et al. Twelve-month prevalence, comorbidity and correlates of mental disorders in Germany: the Mental Health Module of the German Health Interview and Examination Survey for Adults (DEGS1-MH). Int J Meth Psych Res. 2014;3:304–19.
4. Swanson SA, Crow SJ, Le Grange D, Swendsen J, Merikangas KR. Prevalence and correlates of eating disorders in adolescents: results from the national comorbidity survey replication adolescent supplement. Arch Gen Psychiatry. 2011;68(7):714–23.
5. Blinder BJ, Cumella EJ, Sanathara VA. Psychiatric comorbidities of female inpatients with eating disorders. Psychosom Med. 2006;68(3):454–62.
6. Agras WS. Treatment of eating disorders. In: Schatzberg AF, Nemeroff CB, editors. The American Psychiatric Publishing Textbook of Psychopharmacology. 4th ed. Arlington VA: American Psychiatric Association; 2009. p. 1231–40.
7. Woodside DB, Garfinkel PE, Lin E, Goering P, Kaplan AS, Goldbloom DS, et al. Comparisons of men with full or partial eating disorders, men without eating disorders, and women with eating disorders in the community. Am J Psychiatry. 2001;158(4):570–4.
8. Agras WS. The consequences and costs of the eating disorders. Psychiatr Clin North Am. 2001;24(2):371–9.
9. Holtkamp K, Herpertz-Dahlmann B. Zertifizierte Medizinische Fortbildung: Anorexia und Bulimia nervosa im Kindes-und Jugendalter. Dtsch Arztebl. 2005;102(1–2):42.
10. Hilbert A, Pike KM, Goldschmidt AB, Wilfley DE, Fairburn CG, Dohm F-A, et al. Risk factors across the eating disorders. Psychiatry Res. 2014;220(1–2):500–6.
11. Fittig D-PE. Psychosoziale Risikofaktoren. In: Handbuch Essstörungen und Adipositas. Berlin Heidelberg: Springer; 2008. p. 67–74.
12. Jacobi C, Hayward C, de Zwaan M, Kraemer HC, Agras WS. Coming to terms with risk factors for eating disorders: application of risk terminology and suggestions for a general taxonomy. Psychol Bull. 2004;130(1):19–65.
13. Jacobi C, Neubert S. Psychosoziale Risikofaktoren und aufrechterhaltende Bedingungen von Essstörungen. Ein Überblick über den aktuellen Forschungsstand. Psychother in Psychiatrie, Psychother Med Klin Psychol. 2005;10(1):73–86.
14. Keel PK, Forney KJ. Psychosocial risk factors for eating disorders. Int J Eat Disord. 2013;46(5):433–9.
15. Stice E. Risk and maintenance factors for eating pathology: a meta-analytic review. Psychol Bull. 2002;128(5):825–48.
16. Stice E, Ng J, Shaw H. Risk factors and prodromal eating pathology. J Child Psych Psychiatry. 2010;51(4):518–25.
17. Karazsia BT, van Dulmen MH, Wong K, Crowther JH. Thinking meta-theoretically about the role of internalization in the development of body dissatisfaction and body change behaviors. Body Image. 2013;10(4):433–41.
18. Lucas AR, Beard CM, O'Fallon WM, Kurland LT. 50-year trends in the incidence of anorexia nervosa in Rochester, Minn.: a population-based study. Am J Psychiatry. 1991;148(7):917–22.
19. Bundeszentrale für gesundheitliche Aufklärung. Gut drauf. Essstörungen: die heimliche Sucht. 2008. http://www.bzga-essstoerungen.de/index.php?id=165. Accessed 6 Feb 2015.
20. Berger U, Schilke C, Strauss B. Weight concerns and dieting among 8 to 12-year-old children. Psychother Psychosom Med Psychol. 2005;55(7):331–8.
21. Hölling H, Schlack R. Essstörungen im Kindes-und Jugendalter. Bundesgesundheitsblatt-Gesundheitsforschung-Gesundheitsschutz. 2007;50(5–6):794–9.
22. Hawkins N, Richards PS, Granley HM, Stein DM. The impact of exposure to the thin-ideal media image on women. Eat Disord. 2004;12(1):35–50.
23. Presnell K, Bearman SK, Stice E. Risk factors for body dissatisfaction in adolescent boys and girls: a prospective study. Int J Eat Disord. 2004;36(4):389–401.
24. Stice E, Whitenton K. Risk factors for body dissatisfaction in adolescent girls: a longitudinal investigation. Dev Psychol. 2002;38(5):669.
25. Paxton SJ, Neumark-Sztainer D, Hannan PJ, Eisenberg ME. Body dissatisfaction prospectively predicts depressive mood and low self-esteem in adolescent girls and boys. J Clinical Child Adolesc Psychol. 2006;35(4):539–49.
26. Helfert S, Warschburger P. A prospective study on the impact of peer and parental pressure on body dissatisfaction in adolescent girls and boys. Body Image. 2011;8(2):101–9.
27. Stice E, Shaw H. Eating disorder prevention programs: a meta-analytic review. Psychol Bull. 2004;130(2):206.
28. Kiehl K. Risikofaktoren für Essstörungen unter besonderer Berücksichtigung medialer Einflussfaktoren. Dissertation: University of Regensburg; 2011.
29. Taylor CB, Bryson S, Luce KH, Cunning D, Doyle AC, Abascal LB, et al. Prevention of eating disorders in at-risk college-age women. Arch Gen Psychiatry. 2006;63(8):881–8.
30. Stice E, Trost A, Chase A. Healthy weight control and dissonance-based eating disorder prevention programs: results from a controlled trial. Int J Eat Disord. 2003;33(1):10–21.
31. Berger U, Sowa M, Bormann B, Brix C, Strauss B. Primary prevention of eating disorders: characteristics of effective programmes and how to bring them to broader dissemination. Eur Eat Disord Rev. 2008;16(3):173–83.
32. Berger U, Strauß B, Ziegler P. PriMa für Barbie: Formative Evaluation eines Programms zur Primärprävention von Magersucht bei Mädchen ab der sechsten Klasse. Z Psychosom Med Psychother. 2008;54(1):32–45.
33. Bormann B, Brix C, Sowa M, Strauß B. Evaluierte Programme zur Prävention von Essstörungen. Ernahrung Wiss Prax. 2008;2(4):159–68.
34. Stice E, Shaw H, Marti CN. A meta-analytic review of eating disorder prevention programs: encouraging findings. Annu Rev Clin Psychol. 2007;3:207–31.
35. Botvin GJ, Tortu S. Preventing adolescent substance abuse through life skills training. In: Fourteen ounces of prevention: a casebook for practitioners. Washington, DC: American Psychological Association; 1988. p. 98–110.
36. Härter M, Kentgens M, Brandes A, Bock T, Dirmaier J, Erzberger M, et al. Rationale and content of psychenet: the Hamburg Network for Mental Health. Eur Arch Psychiatry Clin Neurosci. 2012;262(2):57–63.
37. Gumz A, Uhlenbusch N, Weigel A, Wegscheider K, Romer G, Löwe B. Decreasing the duration of untreated illness for individuals with anorexia nervosa: study protocol of the evaluation of a systemic public health intervention at community level. BMC Psychiatry. 2014;14(1):300.
38. Deutsche Gesellschaft für Ernährung. Vollwertig essen und trinken nach den 10 Regeln der DGE. 2013. http://www.dge.de/fileadmin/public/doc/fm/10-Regeln-der-DGE.pdf. Accessed 6 Feb 2015.
39. Hilbert A, Buerger A, Hartmann AS, Spenner K, Czaja J, Warschburger P. Psychometric evaluation of the eating disorder examination adapted for children. Eur Eat Disord Rev. 2013;21(4):330–9.
40. Mond JM, Hay PJ, Rodgers B, Owen C, Beumont PJ. Validity of the Eating Disorder Examination Questionnaire (EDE-Q) in screening for eating disorders in community samples. Behav Res Ther. 2004;42(5):551–67.

41. Hilbert A, Tuschen-Caffier B, Karwautz A, Niederhofer H, Munsch S, Eating Disorder Examination- Questionnaire. Evaluation der deutschsprachigen Übersetzung. Diagnostica. 2007;53:144–54.
42. Heinberg LJ, Thompson JK, Stormer S. Development and validation of the Sociocultural Attitudes Towards Appearance Questionnaire. Int J Eat Disord. 1995;17(1):81–9.
43. Knauss C, Paxton SJ, Alsaker FD. Validation of the german version of the Sociocultural Attitudes Towards Appearance Questionnaire (SATAQ-G). Body Image. 2009;6(2):113–20.
44. Childress AC, Brewerton TD, Hodges EL, Jarrell MP. The Kids' Eating Disorders Survey (KEDS): a study of middle school students. J Am Acad Child Adolesc Psychiatry. 1993;32(4):843–50.
45. Childress AC, Jarrell MP, Brewerton TD. The Kids' Eating Disorders Survey (KEDS): internal consistency, component analysis, and reliability. Eat Disord. 1993;1(2):123–33.
46. Rotatori AF. Multidimensional Self Concept Scale. Measurement Evaluation Counsel Dev. 1994;26(4):265–8.
47. Schütz A, Sellin I. Multidimensionale Selbstwertskala: MSWS. Gottingen: Hogrefe; 2006.
48. Gräfe K, Zipfel S, Herzog W, Löwe B. Screening psychischer Störungen mit dem "Gesundheitsfragebogen für Patienten (PHQ-D)". Diagnostica. 2004;50(4):171–81.
49. Löwe B, Unützer J, Callahan CM, Perkins AJ, Kroenke K. Monitoring depression treatment outcomes with the Patient Health Questionnaire-9. Med Care. 2004;42(12):1194–201.
50. Kroenke K, Spitzer RL. The PHQ-9: a new depression diagnostic and severity measure. Psychiatr Annals. 2002;32(9):509–15.
51. Kroenke K, Spitzer RL, Williams JB. The PhQ-9. J Gen Intern Med. 2001;16(9):606–13.
52. Spitzer RL, Kroenke K, Williams JB, Löwe B. A brief measure for assessing generalized anxiety disorder: the GAD-7. Arch Inter Med. 2006;166(10):1092–7.
53. Löwe B, Decker O, Müller S, Brähler E, Schellberg D, Herzog W, et al. Validation and standardization of the Generalized Anxiety Disorder Screener (GAD-7) in the general population. Med Care. 2008;46(3):266–74.
54. Moher D, Hopewell S, Schulz KF, Montori V, Gøtzsche PC, Devereaux PJ, et al. CONSORT 2010 explanation and elaboration: updated guidelines for reporting parallel group randomised trials. J Clin Epidemiol. 2010;63(8):e1–37.

Submit your next manuscript to BioMed Central and take full advantage of:

- Convenient online submission
- Thorough peer review
- No space constraints or color figure charges
- Immediate publication on acceptance
- Inclusion in PubMed, CAS, Scopus and Google Scholar
- Research which is freely available for redistribution

Submit your manuscript at www.biomedcentral.com/submit

BioMed Central
