# Supplementary material for: Efficacy of a prevention program for eating disorders in schools: a cluster-randomized controlled trial
Source: BMC Psychiatry. 2017 Aug 11;17:293. doi: 10.1186/s12888-017-1454-4 (PMC5553667; doi:10.1186/s12888-017-1454-4)
Supplement: Supplementary file 2 — Baseline and follow-up assessment outcomes based on least-squares mean estimates of a mixed-model analysis within the intention-to-treat sample (n = 1020 intervention participants; n = 981 control participants). (DOCX 21 kb) [file 12888_2017_1454_MOESM2_ESM.docx]

**Table S1** Baseline and follow-up assessment outcomes based on least-squares mean estimates of a mixed-model analysis within the intention-to-treat sample (n = 1020 intervention participants; n = 981 control participants)

|  | **Intervention group (n = 1020)** | | | **Control group (n = 981)** | | | **Group*Time**  **Inter-action** *p* | **Between-group differences** | | |
| --- | --- | --- | --- | --- | --- | --- | --- | --- | --- | --- |
|  | Raw mean (SD) | Change to baseline | | Raw mean (SD) | Change to baseline | |  | Adjusted mean difference (95%CI) |  |  |
| **Outcome variables** |  | Adjusted mean (95% CI) | *p* |  | Adjusted mean  (95% CI) | *p* |  |  | *p* | *ES* |
| **Primary outcome*** | | | | | | | | | | |
| **ChEDE** |  |  |  |  |  |  |  |  |  |  |
| Baseline | 0.87 (1.12) |  |  | 0.87 (1.12) |  |  |  |  |  |  |
| Follow-up | 0.87 (1.03) | -0.02 (-0.11; 0.08) | .72 | 0.88 (0.99) | -0.03 (-0.13; 0.08) | 0.58 |  | -0.01 (-0.13; 0.15) | .86 |  |
| **Secondary outcomes**** | | | | | | | | | | |
| **ChEDE** |  |  |  |  |  |  |  |  |  |  |
| Baseline | 0.87 (1.12) |  |  | 0.87 (1.04) |  |  |  |  |  |  |
| Post | 0.87 (1.08) | -0.02 (-0.09; 0.06) | .68 | 0.85 (1.08) | -0.05 (-0.13; 0.04) | 0.26 | .41 | 0.03 (-0.08; 0.15) | .56 |  |
| Follow-up | 0.87 (1.03) | -0.01 (-0.09; 0.07) | .79 | 0.88 (0.99) | -0.02 (-0.10; 0.07) | 0.66 |  | 0.01 (-0.11; 0.12) | .88 |  |
| **Knowledge** |  |  |  |  |  |  |  |  |  |  |
| Baseline | 8.63 (2.98) |  |  | 9.08 (2.72) |  |  |  |  |  |  |
| Post | 11.64 (3.62) | 2.98 (2.41; 3.55) | <.01 | 9.19 (2.98) | 0.18 (-0.49; 0.84) | .58 | <.01 | 2.80 (1.93; 3.68) | <.01 | 0.97 |
| Follow-up | 10.04 (3.37) | 1.38 (0.81; 1.95) | <.01 | 9.42 (3.08) | 0.40 (-0.26; 1.06) | .22 |  | 0.98 (0.11; 1.85) | .03 | 0.38 |
| **SATAQ Internalization** | |  |  |  |  |  |  |  |  |  |
| Baseline | 11.68 (6.07) |  |  | 11.76 (5.73) |  |  |  |  |  |  |
| Post | 12.04 (5.86) | 0.40 (-0.03; 0.84) | .07 | 11.95 (5.56) | 0.18 (-0.31; 0.66) | .45 |  | 0.23 (-0.42; 0.88) | .47 |  |
| Follow-up | 11.97 (5.60) | 0.33 (-0.10; 0.76) | .13 | 12.18 (5.68) | 0.41 (-0.07; 0.90) | .09 | .10 | -0.08 (-0.73; 0.57) | .79 |  |
| **SATAQ Awareness** | |  |  |  |  |  |  |  |  |  |
| Baseline | 13.48 (4.64) |  |  | 13.78 (4.63) |  |  |  |  |  |  |
| Post | 13.68 (5.12) | 0.21 (-0.27; 0.68) | .38 | 13.93 (4.70) | 0.26 (-0.28; 0.80) | .32 |  | -0.05 (-0.67; 0.77) | .88 |  |
| Follow-up | 13.51 (4.80) | 0.04 (-0.44; 0.52) | .87 | 14.03 (4.68) | 0.36 (-0.18; 0.90) | .17 | .18 | -0.32 (-1.05; 0.40) | .35 |  |
| **SATAQ Pressure** | |  |  |  |  |  |  |  |  |  |
| Baseline | 8.98 (4.75) |  |  | 9.11 (4.57) |  |  |  |  |  |  |
| Post | 10.25 (4.77) | 1.30 (1.08; 1.56) | <.01 | 9.80 (4.68) | 0.71 (0.49; 0.93) | <.01 |  | 0.59 (0.28; 0.90) | <.01 | 0.04 |
| Follow-up | 9.77 (4.56) | 0.82 (0.60; 1.04) | <.01 | 10.07 (4.55) | 0.99 (0.77; 1.21) | <.01 | <.01 | -0.17 (-0.47; 0.14) | .30 |  |
| **MSCS general** | |  |  |  |  |  |  |  |  |  |
| Baseline | 111.10 (19.67) |  |  | 111.54 (18.26) |  |  |  |  |  |  |
| Post | 112.29 (18.18) | 1.26 (-0.18; 2.70) | .08 | 113.18 (17.47) | 2.23 (0.65; 3.82) | .01 |  | -0.97 (-3.11; 1.16) | .35 |  |
| Follow-up | 113.42 (18.69) | 2.15 (0.71; 3.59) | .01 | 113.42 (17.94) | 2.48 (0.89; 4.06) | <.01 | .37 | -0.33 (-2.46; 1.81) | .75 |  |
| **MSCS body related** | |  |  |  |  |  |  |  |  |  |
| Baseline | 46.41 (12.25) |  |  | 47.57 (11.63) |  |  |  |  |  |  |
| Post | 46.33 (11.15) | -0.12 (-0.89; 0.66) | .76 | 47.51 (11.14) | 0.60 (-0.24; 1.44) | .15 |  | -1.04 (-2.17; 0.09) | .07 |  |
| Follow-up | 47.18 (11.19) | 0.73 (-0.05; 1.51) | .06 | 48.26 (11.29) | 1.35 (0.51; 2.19) | <.01 | .84 | -0.72 (-1.85; 0.41) | .20 |  |
| **PHQ-9** |  |  |  |  |  |  |  |  |  |  |
| Baseline | 5.53 (4.45) |  |  | 5.52 (4.35) |  |  |  |  |  |  |
| Post | 5.35 (4.47) | -0.08 (-0.41; 0.24) | .60 | 5.42 (4.04) | -0.14 (-0.50; 0.23) | .44 |  | 0.05 (-0.43; 0.54)  0.13 (-0.36; 0.61) | .82  .59 |  |
| Follow-up | 5.41 (4.13) | -0.02 (-0.35; 0.31) | .89 | 5.41 (4.02) | -0.15 (-0.51; 0.22) | .40 | .64 |  |  |  |
| **GAD-7** |  |  |  |  |  |  |  |  |  |  |
| Baseline | 3.94 (4.05) |  |  | 3.91 (3.99) |  |  |  |  |  |  |
| Post | 3.48 (3.84) | -0.37 (-0.67; -0.07) | .02 | 4.10 (3.86) | 0.16 (-0.18;0.49) | .33 |  | -0.52 (-0.97; -0.08) | .02 | 0.11 |
| Follow-up | 3.75 (3.74) | -0.09 (-0.39; 0.21) | .52 | 3.99 (3.82) | 0.05 (-0.29; 0.38) | .77 | <.01 | -0.14 (-0.59; 0.31) | .51 |  |

*Note*. ChEDE-Q = Children’s Eating Disorder Examination Questionnaire; PHQ-9 = Depression Module of Patient Health Questionnaire; GAD-7 = Generalized Anxiety Scale; SATAQ = Social Attitudes of Appearance Questionnaire; MSCS = Multidimensional Self-Concept Scale; general, body related. * Random-effects analysis of covariance. ** Random-effects repeated-measures analysis of covariance. Adjusted mean scores are controlled for BMI, baseline values, sex, grade and ChEDE baseline score.
